# Atomic visualization of a flipped-back conformation of bisected glycans bound to specific lectins

**DOI:** 10.1038/srep22973

**Published:** 2016-03-14

**Authors:** Masamichi Nagae, Mayumi Kanagawa, Kana Morita-Matsumoto, Shinya Hanashima, Yasuhiko Kizuka, Naoyuki Taniguchi, Yoshiki Yamaguchi

**Affiliations:** 1 Structural Glycobiology Team, 2-1 Hirosawa, Wako, Saitama 351-0198, Japan; 2 Department of Chemistry, Osaka University, Machikaneyama, Toyonaka, Osaka 560-0043, Japan; 3 Disease Glycomics Team, Systems Glycobiology Research Group, RIKEN-Max Planck Joint Research Center, RIKEN Global Research Cluster, 2-1 Hirosawa, Wako, Saitama 351-0198, Japan

## Abstract

Glycans normally exist as a dynamic equilibrium of several conformations. A fundamental question concerns how such molecules bind lectins despite disadvantageous entropic loss upon binding. Bisected glycan, a glycan possessing bisecting *N*-acetylglucosamine (GlcNAc), is potentially a good model for investigating conformational dynamics and glycan-lectin interactions, owing to the unique ability of this sugar residue to alter conformer populations and thus modulate the biological activities. Here we analyzed bisected glycan in complex with two unrelated lectins, Calsepa and PHA-E. The crystal structures of the two complexes show a conspicuous flipped back glycan structure (designated ‘back-fold’ conformation), and solution NMR analysis also provides evidence of ‘back-fold’ glycan structure. Indeed, statistical conformational analysis of available bisected and non-bisected glycan structures suggests that bisecting GlcNAc restricts the conformations of branched structures. Restriction of glycan flexibility by certain sugar residues may be more common than previously thought and impinges on the mechanism of glycoform-dependent biological functions.

Glycans, polymers of sugar residues, often adopt several energetically privileged conformations in solution. The array of conformations is likened to a bunch of keys, except that the conformers are in rapid equilibrium under physiological conditions. A cognate protein selects a preferred conformation (or “key”) of the glycan, and binding needs to overcome the unfavorable entropy loss, usually through an enthalpic gain achieved by creating a network of hydrogen bonds^1^,^2^,^3^. Although the functional role of glycan dynamics is not fully understood, it may, as with some intrinsically disordered proteins^4^, be advantageous in attaining several interactions with different protein partners. Statistical analyses of lectin-glycan complex crystal structures indicate that the lectin-bound glycan conformations mostly reflect stable or metastable conformations in solution^5^,^6^,^7^,^8^,^9^. Individual lectins seem to have evolved to recognize/bind each of the more stable conformers of a particular glycan (Fig. 1A).

Biantennary *N*-glycan, the focus of this study, is one of the most common *N*-glycan structures attached to glycoproteins (Fig. 1B) and its conformations have been studied in detail mainly by molecular dynamics (MD) simulations. MD simulations indicate that the introduction of sugar residue bisecting GlcNAc to the branching point of the biantennary glycan shifts the conformational equilibria between extended and folded glycan structures^10^ and can reduce the number of major glycan conformers^11^,^12^. Intriguingly, replica exchange molecular dynamics (REMD) simulations demonstrated that the introduction of a sugar residue bisecting GlcNAc to the branching point of the biantennary glycan dramatically shifts the conformational equilibria, thereby reducing the number of major conformers from five to two. The most populated conformation of bisected *N*-glycan is termed the ‘back-fold’ conformation, in which the α1–6 arm is flipped back towards the stem region of *N*-glycan (GlcNAcβ1–4GlcNAc) (Fig. 1C). This switch-like change attributable to bisecting GlcNAc has also been suggested by NMR-based analysis^13^,^14^,^15^, time-resolved fluorescence resonance energy transfer (FRET) analysis using fluorescently labelled *N*-glycan^16^ and biochemical assays with neoglycoproteins carrying synthetic biantennary *N*-glycans^17^.

Importantly, bisected *N*-glycan may act as a suppressor of cancer metastasis^18^. Modification of bisected *N*-glycan on particular proteins, including epidermal growth factor receptor (EGFR), E-cadherin and integrin, possibly suppress tumor progression and migration^19^,^20^. In particular, a particular *N*-glycan on the β-propeller domain of the integrin α5 subunit is modified with bisecting GlcNAc and regulates its function^21^. The presence of bisecting GlcNAc in an acceptor substrate inhibits several glycosyltransferases, such as GlcNAc transferase V (GnT-V)^22^ and GlcNAc transferase IV (GnT-IV)^23^. Such inhibition may affect biological functions by abolishing the formation of polylactosamine^24^. The introduction of bisecting GlcNAc down-regulates the antigenic α-galactose epitope by modulating the biosynthesis of terminal glycan structures^25^ and even modulates the binding of antibody to Fc receptor^26^. Bisecting GlcNAc also modulates the intracellular localization of carrier glycoproteins. A Recent study showed that the addition of bisecting GlcNAc to β-site amyloid precursor protein cleaving enzyme-1 (BACE1) blocks the targeting of this protein to the lysosome in Alzheimer’s disease^27^, although the mechanism is not fully understood. Consequently, considerable attention has been paid to the relationship between its conformations and biological activities. In line with this aspect, we revealed the first crystal structure of a bisected *N*-glycan unit in complex with murine dendritic cell inhibitory receptor 2 (mDCIR2)^28^. The mDCIR2 binds the bisected *N*-glycan unit in an extended form. We also crystallized the same bisected *N*-glycan unit in complex with phytohemagglutinin from *Phaseolus vulgaris* (PHA-E)^29^. However, the conformation of PHA-E-bound bisected glycan unit could not be determined because only the disaccharide unit (GlcNAcβ1–2Man) gave electron density. Hence the back-fold conformation of bisected *N*-glycan in complex with lectin has not yet been observed through crystallographic analysis.

Our present study is based on the idea that each three-dimensional crystal structure of protein-glycan complex provides a static snapshot of a particular glycan conformer selected from multiple conformations, and an accumulation of snapshots informs on the most stable and metastable conformations^30^. In an attempt to obtain such “snapshots” of biantennary *N*-glycan, we selected for crystallographic studies two unrelated plant lectins, Calsepa from *Calystegia sepium* and PHA-E from *Phaseolus vulgaris*, which show 4- to 5-fold increase in affinity toward bisected biantennary *N*-glycans compared with control non-bisected glycan^31^,^32^.

## Results

### Binding specificity of Calsepa and PHA-E lectins to bisected glycans

**Figure 1:**
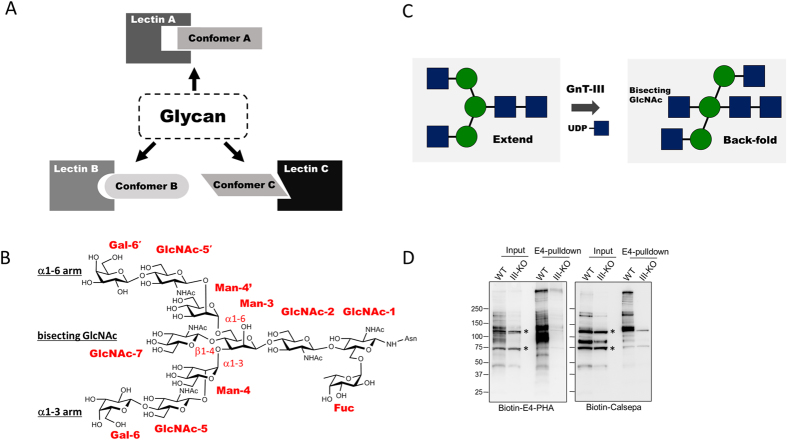
Multiple glycan conformations and unique selection by individual lectins. (**A**) The working hypothesis of this study. A flexible glycan assumes various stable or metastable conformations (e.g. conformation **A–C**), and each lectin (e.g. lectin **A–C**) selects one specific conformation among them. (**B**) Representative chemical structure of a bisected biantennary complex-type *N*-glycan. Carbohydrate residues and each glycosidic linkage are labeled in red. (**C**) Chemical modification of bisecting *N*-acetylglucosamine (GlcNAc) catalyzed by GnT-III. Two major conformations, extend and back-fold, are shown. (**D**) Detection of bisected glycans in mice brain extracts by Calsepa and PHA-E lectins. Proteins were extracted from brain membrane fractions of 20-week-old wild-type or *Mgat3*-defiicient mice, and then incubated with E4-PHA-beads. The membrane extracts (input) and proteins bound to the beads (E4-pulldown) were blotted with E4-PHA lectin or Calsepa lectin. Asterisks indicate avidin-reactive non-specific bands.

PHA-E is a legume lectin (120 kDa as tetramer) which is widely used for detecting bisected glycans^29^,^33^. Calsepa lectin (16 kDa) belongs to mannose-binding type Jacalin-related lectin^34^, and was found to show relatively higher affinity toward bisected glycans than non-bisected glycans^31^. In order to confirm the binding ability of these lectins toward bisected glycans, we performed binding experiments using mouse brain samples, which are known to highly express bisected glycans^35^. Pull-down experiments using immobilized PHA-E lectins showed that several distinct proteins were stained with biotinylated PHA-E (Fig. 1D, left panel). Importantly, most of these PHA-E-bound proteins were also stained with biotinylated Calsepa lectin (Fig. 1D, right panel). These bands were not detected in a brain sample from knockout mice for GlcNAc transferase III (GnT-III, encoded by *Mgat3* gene), which is the sole enzyme essential for the synthesis of bisected glycans in mammals^36^,^37^. It indicates that Calsepa lectin has the ability to bind to bisected glycans, and therefore Calsepa lectin has potential to be used as a scaffold for X-ray crystallographic and NMR analyses of bisected glycans.

### Bisected glycan is trapped in a back-fold conformation in association with Calsepa lectin

**Figure 2:**
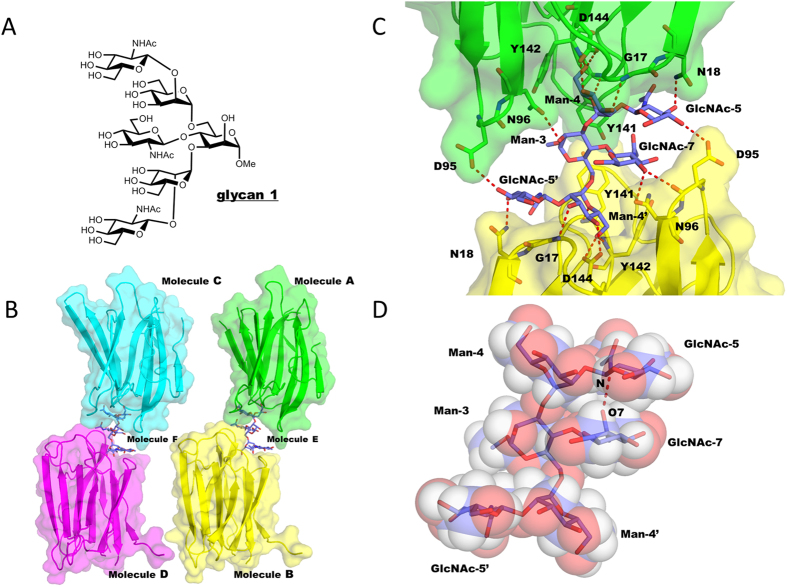
Two Calsepa molecules sandwich bisected hexasaccharide (glycan 1) in a back fold conformation. (**A**) Chemical structure of bisected glycan (glycan 1) used for Calsepa complex. (**B**) Overall structure of Calsepa in complex with glycan 1 in the asymmetric unit. (**C**) Close up view of sandwiched glycan 1. Potential hydrogen bonds between Calsepa and glycan 1 are indicated with red dotted lines. (**D**) The structure of Calsepa-bound glycan 1 shown in rod and semi-transparent sphere models. Possible hydrogen bond between GlcNAc-5 and GlcNAc-7 is shown in a red dotted line.

To obtain the atomic details of lectin-bound bisected glycan, the crystal structure of Calsepa in complex with GlcNAc-terminated bisected *N*-glycan unit (glycan 1 in Fig. 2A) was determined at 1.85 Å resolution (Table 1). The asymmetric unit contains four Calsepa molecules and two bisected glycans (Fig. 2B). Two Calsepa molecules sandwich one bisected glycan via an extensive hydrogen bond network as listed in Table S1. One Calsepa molecule interacts with Man-3, Man-4, GlcNAc-5 and GlcNAc-5′ while the other Calsepa molecule binds to GlcNAc-5, Man-4′, GlcNAc-5′ and GlcNAc-7 (bisecting GlcNAc) (Fig. 2C, the numbering of each sugar residue is indicated in Fig. 1B), indicating that these two Calsepa molecules bind to the bisected glycan in distinct ways. The sandwich-like 2:1 lectin-glycan interaction seems to stabilize the glycan conformation firmly.

Since we performed X-ray crystallographic analysis using *N*-glycan without a chitobiose moiety, we modeled the whole bisected *N*-glycan into the crystal structure of Calsepa lectin complex (Fig. S1). In the model building, the bisected glycan derived from a crystal structure of glycosylated catrocollastatin/vascular apoptosis-inducing protein^38^ (PDB code 2DW2) was superimposed onto the crystal structure of truncated bisected glycan. The modeled structure indicates that the chitobiose moiety is accommodated in the crystal lattice without significant steric hindrance. In the model, GlcNAc-5′ in the 1–6 branch is in contact with GlcNAc-2 in the chitobiose moiety, suggesting that this inter-branch interaction may occur in intact bisected *N*-glycan.

The 3D structure of glycan 1 is defined by the dihedral angles of five distinct glycosidic linkages as listed in Table S2. The conformations of the two glycans in the asymmetric unit are almost same; therefore molecule E is described as representative hereafter. The global conformations of bisected and non-bisected biantennary glycans are defined by three dihedral angles (*ϕ*_α1–6_, *φ*_α1–6_, *ω*_α1–6_) of Manα1–6Man linkage, and five distinct conformations have been proposed by REMD simulations: half back fold (*ϕ*_α1–6_ = 70, *φ*_α1–6_ = 60, *ω*_α1–6_ = 60), tight back fold (*ϕ*_α1–6_ = 70, *φ*_α1–6_ = 60, *ω*_α1–6_ = 180), back fold (*ϕ*_α1–6_ = 70, *φ*_α1–6_ = 90, *ω*_α1–6_ = 60), extend-a (*ϕ*_α1–6_ = 70, *φ*_α1–6_ = 180, *ω*_α1–6_ = 60) and extend-b (*ϕ*_α1–6_ = 70, *φ*_α1–6_ = 180, *ω*_α1–6_ = 180)^11^. In the Calsepa-glycan complex, the α1–6 branch of the glycan is flipped backward (*ϕ*_α1–6_ = 95, *φ*_α1–6_ = 105, *ω*_α1–6_ = 58) and the conformation is classified as back-fold. This conformation has been predicted to be a major conformation of bisected glycans by REMD simulations^11^,^12^. Two GlcNAc residues, GlcNAc-5 and GlcNAc-7, are aligned in parallel and the putative hydrogen atoms (H2 and H4) of GlcNAc-7 can make van der Waals contact with the hydrogen atoms (H1, H3 and H5) of GlcNAc-5 (Fig. 2D). In addition, one hydrogen bond is observed between NH group of GlcNAc-5 and *N*-acetyl carbonyl oxygen atom of GlcNAc-7. These intramolecular glycan interactions seem to stabilize the glycan conformation, acting together with the lectin-glycan interactions.

The back-fold conformation seems essential to attain the sandwich-like 2:1 interaction. For example, if one Calsepa molecule binds bisected glycan in an extend-b conformation (*ϕ*_α1–6_ = 70, *φ*_α1–6_ = 180, *ω*_α1–6_ = 180)^11^, binding of a second Calsepa is unlikely without steric clashes between proteins (Fig. S2A). In summary, Calsepa lectin can be considered ‘back-fold conformer selective’, and will not favor a glycan with an extend conformation.

### NMR analyses provide evidence of back-fold conformation in solution

**Figure 3:**
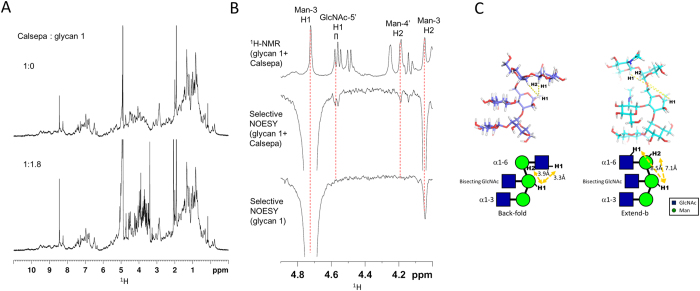
Back fold conformation of glycan 1 as evidenced by solution NMR analysis. (**A**) Titration study monitored by ^1^H-NMR spectra. Protein-to-ligand ratio is indicated for each NMR spectrum. (**B**) Analysis of the lectin-bound conformation by TR-NOE analysis. 1D ^1^H-NMR spectrum of Calsepa-glycan 1 mixture (1:10 molar ratio) (top) and 1D selective NOESY spectra of glycan 1 in the presence (middle) and absence (bottom) of Calsepa. (**C**) Schematic representation of proton-proton distances between Man-3 H1 and GlcNAc-5′ H1 and between Man-3 H1 and Man-4′ H2. Structures of back-fold and extend-b conformations are derived from Calsepa complex (blue) and mDCIR2 complex (PDB code: 3VYK, cyan), respectively. The distances of the two hydrogen atoms are indicated with yellow dotted lines. Hydrogen atoms are generated with PyMOL.

The crystal structure shows that two Calsepa lectins bind to one bisected glycan in two binding modes. To examine the ligand binding and subsequent oligomer formation in solution, we performed a titration experiment by solution ^1^H-NMR spectroscopy (Fig. 3A). Upon titration of bisected glycan (glycan 1) to the Calsepa lectin solution, chemical shift changes of the protein signals were very small and an increase of line width was not observed, making it difficult to accurately estimate the dissociation constant. These observations suggest that the glycan 1 binds to Calsepa lectin very weakly, and that the sandwich-like 2:1 complex was not formed significantly in solution under the condition tested. A comparison of apo- and ligand-bound Calsepa structures revealed that the structures are almost identical with backbone RMSD values of 0.39~0.75 angstroms (Fig. S3). This means that ligand binding does not induce a conformational change in Calsepa and therefore the chemical shift changes are expected to be very small. The dissociation constant of intact bisected biantennary glycan to Calsepa was reported to be 9.2 μM^31^. The much weaker affinity of glycan1 is likely due to the lack of the chitobiose portion (GlcNAcβ1–4GlcNAc) in the glycan 1 structure. It is possible that both intra- and inter- glycan interactions of the chitobiose moiety contribute to the affinity (Fig. S1).

We turned to transferred nuclear Overhauser enchancement (TR-NOE) analysis for information on the lectin-bound conformation. One dimensional selective NOESY experiments were conducted for glycan 1-Calsepa mixture (glycan 1:Calsepa = 10:1 molar ratio) by inverting the Man-3 H1 signal. The NOE build-up curve shows linearity up to 200 ms for the glycan 1-Calsepa mixture and to 500 ms for glycan 1 alone (Fig. S4). A mixing time of 200 ms yielded strong intra-residue TR-NOE from Man-3 H1 to Man-3 H2 (Fig. 3B). Very unusually, long-range TR-NOEs were observed from Man-3 H1 to GlcNAc-5′ (α1–6 branch) H1 and to Man-4′ H2 signals. Glycan 1 alone gave only a few weak intra-residue NOE signals (Fig. 3B). The TR-NOE result is compatible with the distances of corresponding proton pairs seen in the structure with a back-fold conformation. In the back-fold conformation, the distance between Man-3 H1 and GlcNAc-5′ H1 is 3.3 Å and that between Man-3 H1 and Man-4′ H2 is 3.9 Å (Fig. 3C). Furthermore, the TR-NOE result is completely incompatible with the extend-b conformation, which we have observed in a complex of glycan 1 with another bisected glycan specific lectin DCIR2. In DCIR2-glycan 1 complex, the distance between Man-3 H1 and GlcNAc-5′ H1 is 8.5 Å, and that between Man-3 H1 and Man-4′ H2 is 7.1 Å. We conclude that the glycan assumes a back-fold conformation in solution, rather than an extend-b conformation, when it binds to Calsepa lectin.

### Bisected N-glycan bound to PHA-E lectin is also in a back-fold conformation

**Figure 4:**
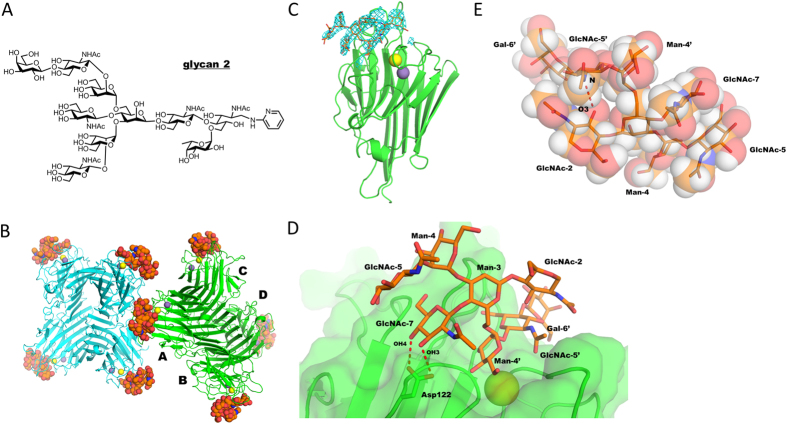
Bisected glycan (glycan 2) shows a flip back conformation in complexed with PHA-E. (**A**) Chemical structure of bisected glycan (glycan 2) used for PHA-E complex. (**B**) Overall structure of PHA-E in complex with glycan 2. Eight protein subunits and eight glycan molecules in the asymmetric unit are shown in ribbon and sphere models, respectively. Calcium and manganese ions are shown in yellow and purple spheres, respectively. (**C**) Monomeric structure (Molecule A) of PHA-E in complex with glycan 2. Omit map contoured at 2.5 σ level around ligand-binding site is shown in cyan mesh. (**D**) Close up view of the ligand binding site. Putative hydrogen bonds with bisecting GlcNAc are shown in red dotted lines. (**E**) The structure of PHA-E-bound glycan 2 depicted in rod and semi-transparent sphere models. The intra-carbohydrate hydrogen bond between GlcNAc-2 and GlcNAc-5′ is shown as a red dotted line.

We have previously reported on a crystal structure of PHA-E in complex with a GlcNAc-terminated bisected *N*-glycan unit (glycan 1). Unfortunately, electron density was observed for only two sugar residues from one branch, which made it impossible to decipher the conformation of the whole glycan^29^. We have now determined the crystal structure of PHA-E in complex with a longer bisected *N*-glycan derivative (glycan 2 in Fig. 4A) at 3.0 Å resolution (Table 1). Eight PHA-E subunits and eight bisected glycan molecules are packed in the asymmetric unit (Fig. 4B). PHA-E forms a tetramer and each monomer binds to one bisected glycan in its primary binding site. The quality of electron density was rather poor yet the electron density was successfully interpreted. The bisected *N*-glycan (glycan 2) was divided into fragments (Manα1–6 branch, core mannose (Man-3), bisecting GlcNAc, Manα1–3 branch, and chitobiose unit), and the trisaccharide unit (Galβ1–4GlcNAcβ1–2Man) was initially fitted using the high resolution crystal structure of a monogalactosylated biantennary *N*-glycan unit in complex with PHA-E^29^. Then the remaining glycan fragments were fitted to the corresponding electron density map in a step-by-step manner. The structure contains eight carbohydrate residues out of ten (Fig. 4C). The α1–6 branch and bisecting GlcNAc interact with PHA-E, while the α1–3 branch and chitobiose unit are exposed to solvent. The only hydrogen bonds are between two hydroxyl groups, OH3 and OH4 of GlcNAc-7 and the side chain of Asp122 in the loop B region (Fig. 4D).

The structure of the octasaccharide part is defined by the dihedral angles of each glycosidic linkage. The dihedral angles of the eight glycan molecules (A-H) in the asymmetric unit are shown in Table S2 and found to be essentially identical. The bisected glycan of molecule A is described as representative hereafter. The three dihedral angles (*ϕ*_α1–6_, *φ*_α1–6_, *ω*_α1–6_) of the Manα1–6Man linkage are (*ϕ*_α1–6_ = 55, *φ*_α1–6_ = 126, *ω*_α1–6_ = 63), showing that the PHA-E-bound bisected glycan is also in a back-fold conformation. The intra-carbohydrate interactions are also observed in bisected glycan of the PHA-E complex (Fig. 4E). The putative H2 and H4 hydrogen atoms of GlcNAc-7 can make van der Waals contact with H1, H3 and H5 hydrogen atoms of GlcNAc-5 of the α1–3 branch. Moreover, the nitrogen atom of GlcNAc-5′ in the α1–6 branch is located within hydrogen bond distance of OH3 of GlcNAc-2 in the chitobiose unit. As in the case of Calsepa, the back-fold conformation appears critical for simultaneous recognition of both the α1–6 branch and GlcNAc-7 (Fig. S2B). Such extensive lectin-glycan interaction cannot be attained with the glycan in an extend-b conformation. Thus both proteins, PHA-E as well as Calsepa, are specifically ‘back-fold conformer selective’, rather than broad bisecting GlcNAc-selective lectins.

### Statistical analysis of bisected and non-bisected glycan conformations

**Figure 5:**
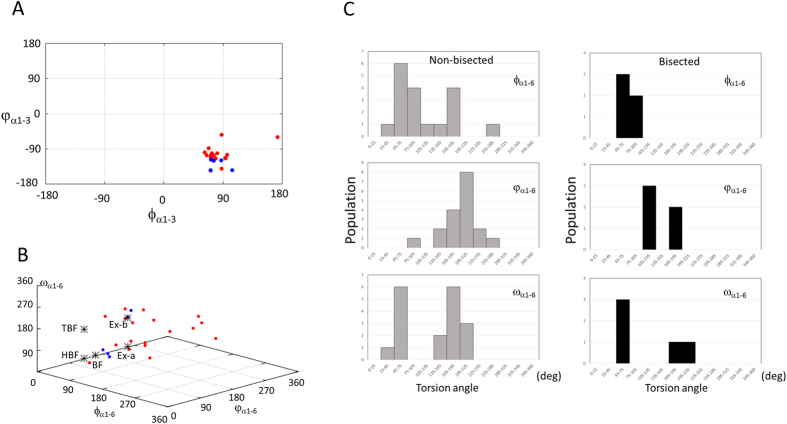
Comparative analysis of bisected and non-bisected biantennary glycans using PDB data. (**A**) Plot of dihedral angles of Manα1–3Man linkage. The *ϕ*_α1–3_ and *φ*_α1–3_ are plotted in horizontal and vertical axes, respectively. The bisected and non-bisected biantennary glycans are plotted as blue and red circles, respectively. (**B**) Plot of dihedral angles of Manα1–6Man linkage. For clarity, the *ϕ*_α1–6_, *φ*_α1–6_ and *ω*_α1–6_ are plotted from 0° to 360° instead of −180° to 180°. Conformations of non-bisected and bisected glycans are plotted in red and blue, respectively. Five proposed conformations, extend-a (Ex-a), extend-b (Ex-b), back-fold (BF), half back-fold (HBF) and tight back-fold (TBF) are indicated with asterisks. (**C**) Histograms of three dihedral angles of Manα1–6Man linkage. The bisected and non-bisected biantennary glycans are shown in right and left panels, respectively.

Our structural study describes for the first time two examples of the flipped back conformation in lectin-glycan complexes. Although statistical conformational analyses of *N*-glycans have been reported^5^,^6^,^7^, they do not focus on the conformational differences between bisected and non-bisected biantennary *N*-glycans. We have now constructed a dataset of biantennary *N*-glycans to investigate the ‘dynamic’ range of bisected and non-bisected biantennary glycans from the Protein Data Bank (Table S3). All data contain GlcNAc-terminated branches, and these are defined by the following glycosidic linkages, GlcNAcβ1–2Man of two branches, Manα1–3Man, Manα1–6Man, and GlcNAcβ1–4Man. The dihedral angles of Manα1–3Man and Manα1–6Man linkages from bisected and non-bisected glycans are plotted in Fig. 5, with those obtained from MD simulations indicated with asterisks^11^.

The average dihedral angles of the Manα1–3Man unit with non-bisected glycans (*ϕ*_α1–3_ = 83 ± 24, *φ*_α1–3_ = −105 ± 22) are similar to those of bisected glycans (*ϕ*_α1–3_ = 82 ± 12, *φ*_α1–3_ = −129 ± 13) (Fig. 5A). These values are also similar to the averaged value obtained from individual glycosidic linkages analysis (*ϕ*_α1–3_ = 72 ± 9, *φ*_α1–3_ = −121 ± 17)^6^. These results indicate that bisecting does not significantly affect the conformational property of this Manα1–3Man unit in biantennary *N*-glycans. However, it should be noted that some outliers were found in non-bisected glycans (see Discussion).

In order to classify the global conformation of the *N*-glycan, three dihedral angles of Manα1–6Man linkage, *ϕ*_α1–6_, *φ*_α1–6_ and *ω*_α1–6_ were utilized (Table S3). In non-bisected biantennary glycans, 10 glycans belong to extend-a or extend-b conformations and only one is classified into the back-fold conformation. The other 9 entries cannot be categorized into any of the predicted conformations. The conformational ensemble of non-bisected biantennary glycans compare favorably with a recent NMR study using pseudo contact shifts, in which extend-a (extended *gg*, 45%) and extend-b (extended *gt,* 35%) are major conformations^39^. In bisected glycan datasets, three entries belong to the back-fold conformation and the other two are extend-b conformations. In general, the three dihedral angles of non-bisected glycans are more broadly distributed compared with bisected glycans (Fig. 5B), which seems to imply that the bisecting GlcNAc restricts the freedom of the Manα1–6Man linkage.

The population histograms of these three dihedral angles are shown in Fig. 5C. The *ϕ*_α1–6_ angles of bisected glycans (*ϕ*_α1–6_ = 73 ± 15) converge in a narrow range while the dihedral angles from non-bisected glycan are more widely distributed. The *ω*_α1–6_ torsion angle adopts three possible staggered rotamers, referred to as *gauche*-*trans* (*gt*, *ω*_α1–6_ = 180°), *gauche*-*gauche* (*gg*, *ω*_α1–6_ = 60°) and *trans*-*gauche* (*tg*, *ω*_α1–6_ = 300° (−60°)). *Gg* and *gt* rotamers, but not *tg*, are observed in both non-bisected and bisected glycans.

## Discussion

**Figure 6:**
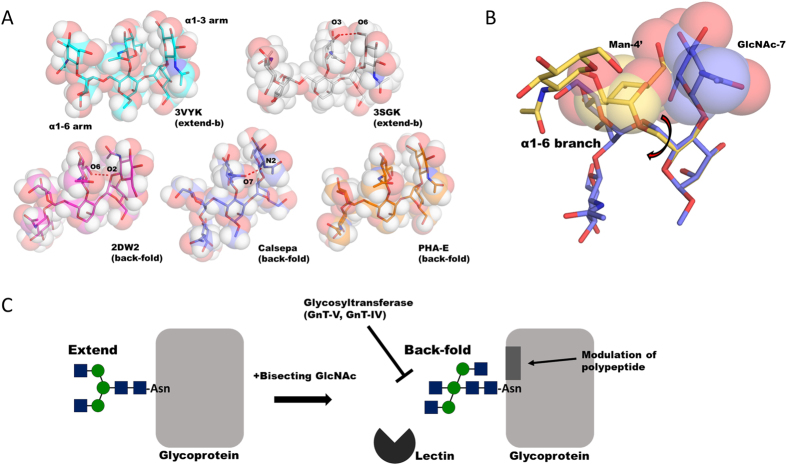
Structural basis of bisected glycan and potential impact on biological function. (**A**) Five bisected glycans are shown in rod and semi-transparent space filling models. Three hydrogen bond patterns between bisecting GlcNAc and adjacent α1–3 branch observed in three bisected glycans (VAP 2B (PDB code: 2DW2), IgG Fc domain (PDB code: 3SGK) and Calsepa) are depicted in red dotted lines. Hydrogen atoms are generated with PyMOL. (**B**) Structural superposition of non-bisected glycan in extend-a conformation (PDB code: 2ARX, orange) and bisected glycans in back-fold conformation (Calsepa complex, blue). Two glycans are shown in rod model. The Man-4′ of non-bisected glycan and bisecting GlcNAc residue are highlighted in semi-transparent sphere model. The Manα1–6Man linkage is indicated by arrow. (**C**) Possible functional roles of bisected glycan deduced from the unique conformational shift. Bisecting GlcNAc regulates the enzymatic activities of various glycosyltransferases such as GnT-IV, GnT-V as well as lectin binding. The conformational shift can also affect the structure of polypeptide near the *N*-glycosylation site.

The interaction between bisecting GlcNAc and the α1–3 branch of a glycan was suggested by NMR analysis and REMD simulation^12^,^13^,^14^,^15^,^40^. Our present structural comparisons among five bisected glycans reveal that the B-face (composed of H2 and H4) of bisecting GlcNAc (GlcNAc-7) preferentially makes van der Waals contact with A-face (composed of H1, H3 and H5) of GlcNAc-5 in the Manα1–3 branch (Fig. 6A). Moreover, three distinctive hydrogen bond patterns between GlcNAc-7 and the Manα1-3 branch are observed (red dotted lines in Fig. 6A). Importantly, the extend-a conformation is not found in the bisected glycan structures. This is likely due to a possible severe steric clash between Man-4′ (α1–6 branch) and bisecting GlcNAc (Fig. 6B). Thus bisecting GlcNAc limits the freedom of the α1–6 branch by “repelling” Man-4′. Taken together, it appears that the back-fold conformation is likely to be induced by favorable van der Waals contacts and a hydrogen bond between Manα1–6 branch and the chitobiose unit, and by an unfavorable steric clash between Man-4′ and bisecting GlcNAc formed in the extend-a conformation (Fig. 4D).

We found that non-bisected glycan adopts some unusual glycosidic conformations of the Manα1-3Man linkage, both in complex with galectin-1 (1SLC_354, (*ϕ*_α1–3_ = 88, *φ*_α1–3_ = −54)) and Zinc-α_2_-glycoprotein (1ZAG_A, (*ϕ*_α1–3_ = 173, *φ*_α1–3_ = −60)). In these conformations, the position of GlcNAc-5 in the α1-3 branch is distant from the position of a putative bisecting GlcNAc, and interaction between bisecting GlcNAc and Manα1-3 branch is not possible (Fig. S5). These observations point to the inherent flexibility of non-bisected glycans, and their ability to occasionally adopt extreme conformations.

The switch-like attribute of bisecting GlcNAc may underlie some physiological effects (Fig. 6C). GnT-V, which adds a GlcNAc residue at Man-4′ via a β1–6 linkage, is inhibited by the presence of bisecting GlcNAc in the acceptor glycan^22^. Likewise, the reaction catalyzed by GnT-IV, which introduces a GlcNAc at Man-4 via a β1–4 linkage, is also prevented by bisecting GlcNAc in the acceptor glycan^23^. The dominant flipped-back conformation of the Manα1–6 branch may be responsible for the inhibition of *N*-glycan branching exhibited by these enzymes.

It is possible that bisecting GlcNAc not only affects the activities of various glycosyltransferases but also modulates the function or intracellular localization of carrier glycoproteins. The addition of bisecting GlcNAc to the *N*-glycan of α5β1 integrin significantly affects the fibronectin-mediated cell adhesion and migration^21^. Perhaps the specific back-fold conformation of bisected glycan affects the adjacent polypeptide part of α5β1 integrin, leading to the modulation of fibronectin binding. It was recently demonstrated that modification of bisecting GlcNAc of BACE1 blocks its lysosomal targeting in Alzheimer’s disease^27^. An endogenous lectin-like molecule may interact with bisected glycan on BACE1 in a conformation-specific manner and determine its localization.

In summary, our study provides the first atomic structures of a flipped-back conformation of bisected *N*-glycan bound to two specific lectins. Other lectins may be profitably used to trap further dominant conformational states and add to our knowledge of the mechanism of yet unknown conformer-dependent regulatory interactions observed in various biological interactions.

## Methods

### Materials

Recombinant Calsepa and PHA-E lectins purified from *Phaseolus vulgaris* were purchased from Wako Pure Chemicals and J-Oil Mils, Inc, respectively. The hexasaccharide, GlcNAcβ1–2Manα1–3[GlcNAcβ1–4][GlcNAcβ1–2Manα1–6]Manα1-*O*-methyl (glycan 1 in Fig. 2A), was chemically synthesized as reported previously^41^. The mono-galactosylated biantennary complex-type *N*-glycan derivative harboring bisecting GlcNAc, GlcNAcβ1–2Manα1–3[GlcNAcβ1–4][Galβ1–4GlcNAcβ1–2Manα1–6]Manβ1–4GlcNAcβ1–4[Fucα1–6]GlcNAc-pyridylamino (PA) (glycan 2 in Fig. 4A), was prepared from sheep immunoglobulin G as described previously^29^.

### Mutant mice

GnT-III (*Mgat3*)-deficient mice were generated as described previously^42^ and kindly provided by Dr. Jamey D. Marth (University of California-Santa Barbara). All animal experiments were approved by the Animal Experiment Committee of RIKEN. The methods were carried out in accordance with approved guidelines.

### Preparation of brain membrane fraction and PHA-E pulldown

Brains from 20-week-old male mice were homogenized with 7 volumes of Tris-buffered saline (TBS) containing protease inhibitors (Roche) using a Potter-type homogenizer. Homogenates were ultracentrifuged at 105,000 × *g* for 30 min at 4 °C, and the resultant pellet was used as a membrane fraction. The membrane fractions were solubilized with TBS containing 0.5% Nonidet P-40, and then centrifuged at 105,000 × *g* for 15 min at 4 °C. The supernatant (input) was incubated with E4-PHA-agarose (J-Oil Mills) for 1 h at 4 °C with gentle shaking. The beads were washed twice with TBS containing 0.1% Nonidet P-40, and then bound proteins were eluted by boiling with SDS sample buffer.

### SDS-PAGE and lectin blot

Proteins were separated by 5–20% SDS-PAGE and then blotted to nitrocellulose membranes. Membranes were blocked with TBS containing 0.1% Tween 20 for 30 min at room temperature and then incubated with biotinylated E4-PHA lectin (Seikagaku Corporation) or Calsepa lectin (US Biological) that had been diluted with TBS containing 0.1% Tween 20, followed by incubation with HRP-avidin (VECTASTAIN ABC Standard Kit). Signals were detected with Western Lightning ECL Pro (PerkinElmer) using ImageQuant LAS-4000mini (GE Healthcare).

### Crystallization, data collection and structure determination

Calsepa in phosphate-buffered saline (pH 7.4) was concentrated up to 5 mg/ml using Amicon Ultra (molecular weight cut off 3K). The bisected hexasaccharide (glycan 1) was mixed with Calsepa solution at a final concentration of 1 mM (three times excess of protein). Freeze-dried PHA-E was dissolved at 9 mg/ml in 20 mM Tris-HCl (pH 8.0) containing 100 mM NaCl. The galactosylated bisected glycan (glycan 2) was mixed with PHA-E solution at a final concentration of 0.6 mM. All crystals were obtained by the sitting drop vapor diffusion method using 0.8-μl drops containing a 50:50 (v/v) mix of protein and reservoir solution at 20 °C. The crystallization conditions were determined by screening using Index (Hampton Research). Crystals of the Calsepa-glycan 1 complex and PHA-E-glycan 2 complex were grown in a reservoir containing 0.1 M HEPES-NaOH (pH 7.5) and 3.0 M NaCl (Index #11) and 0.1 M sodium acetate (pH 4.5) and 25% (w/v) PEG3,350 (Index #41), respectively. Before X-ray diffraction experiments, crystals were soaked in the reservoir solution containing 25% (v/v) ethylene glycol and flash-cooled in nitrogen gas stream at 95 K. X-ray diffraction data sets for the crystals were collected at the synchrotron radiation source at BL-5A and AR-NW12A in the Photon Factory (Tsukuba, Japan). The diffraction images were processed and scaled using HKL2000 program packages^43^. Phase determinations were performed by the molecular replacement method with an unliganded form of Calsepa (PDB code 1OUW^34^) and PHA-E-ligand complex (PDB code 3WCS^29^) using MOLREP^44^. Further model building was performed manually using the program COOT^45^. Refinement was conducted using REFMAC5^46^. The stereochemical quality of the final models was assessed by MolProbity^47^. Data collection and refinement statistics are summarized in Table 1. All figures were prepared with PyMOL (DeLano Scientific). The structure factors and coordinates of Calsepa-bisected glycan (glycan 1) complex and PHA-E-bisected glycan (glycan 2) complex are deposited in Protein Data Bank under the accession codes 5AV7 and 5AVA, respectively.

### Solution NMR experiments

All the NMR spectra were recorded either with a 600 MHz spectrometer (Bruker BioSpin) equipped with a 5-mm TXI probe or a 500 MHz spectrometer (BrukerBiospin) with a 5-mm TXI cryogenic probe. Probe temperature was set at 15 °C. Chemical shifts are shown in parts per million (ppm), adjusted at 0 ppm with 4,4-dimethyl-4-silapentane-1-sulfonic acid (DSS). Calsepa lectin (0.17 mM) in phosphate-buffered saline (pH 7.4) was mixed with bisected glycan in 50 mM Tris-HCl, 100 mM NaCl, 1% (w/v) NaN_3_, pH 8.0, yielding the protein to ligand molar ratio of 1:10 for transferred-NOESY experiment. For the titration experiment, Calsepa lectin (0.06 mM) in 20 mM sodium phosphate and 50 mM NaCl was mixed with bisected glycan solution with a protein to ligand molar ratio from 1:0.3 to 1:1.8. Bisected glycan solution (0.5 mM) without Calsepa lectin was used for the control NMR experiments. All the NMR samples were dissolved in 99.9% D_2_O.

Selective 1D NOESY spectra of bisected glycan in the presence of Calsepa were collected with mixing times of 50, 100, 150, 200, 300, 400 and 500 ms. Man-3 H1 signal was selectively inverted with a 180° Gaussian shaped pulse of 120 ms or a rectangular 40-ms pulse. The data were collected in 16 k data points with the spectral width of 7,500 Hz. Selective 1D NOESY spectra of bisected glycan without protein were collected with mixing times of 100, 200, 300, 400 and 500 ms under the same conditions.

### Statistical analysis of non-bisected and bisected biantennary glycans deposited in Protein Data Bank

Coordinates of non-bisected and bisected biantennary glycans were extracted from the Protein Data Bank (PDB) as of March 2015. The extracted glycan structures met the criteria of containing the core *N*-glycan branching unit GlcNAcβ1–2Manα1–3[GlcNAcβ1–2Manα1–6]Man with or without bisecting GlcNAc. To avoid redundancy within the dataset, the following points were considered: When more than two conformers are present in the asymmetric unit, one conformer is carefully selected and the other glycan structures omitted. Several entries of non-bisected biantennary glycans were carefully omitted due to wrong connections between the carbohydrate residues. Since 39 structures of IgG Fc domains with non-bisected biantennary glycans have been deposited, the highest resolution structure (PDB code: 1L6X) was selected as representative of Fc glycan structures. In the end, the dataset includes 18 structures of non-bisected biantennary glycans and 5 bisected glycans as listed in Table S3. The non-bisected biantennary glycan dataset includes 12 glycans from lectin or antibody complexes and 6 glycans from glycoproteins. The bisected *N*-glycan dataset is composed of 5 entries, three of them are derived from lectin complex structures including the structures obtained in this study and the other two are from glycan moieties of glycoproteins. The dihedral angles of the glycosidic linkages were calculated using a program PyMOL and plotted using gnuplot. The definitions of dihedral angles of glycosidic linkages used in this study are as follows: Manα1-3Man (*ϕ*_α1–3_ = O5-C1-O-C3′, *φ*_α1–3_ = C1-O-C3′-C2′), Manα1-6Man (*ϕ*_α1–6_ = O5-C1-O-C6′, *φ*_α1–6_ = C1-O-C6′-C5′, *ω*_α1–6_ = O-C6′-C5′-C4′), GlcNAcβ1-4Man (*ϕ*_β1–4_ = O5-C1-O-C4′, *φ*_β1–4_ = C1-O-C4′-C3′), GlcNAcβ1-2Man (*ϕ*_β1–2_ = O5-C1-O-C2′, *φ*_β1–2_ = C1-O-C2′-C1′). The conformation of biantennary glycan is classified into half back fold (*ϕ*_α1–6_ = 70, *φ*_α1–6_ = 60, *ω*_α1–6_ = 60), tight back fold (*ϕ*_α1–6_ = 70, *φ*_α1–6_ = 60, *ω*_α1–6_ = 180), back fold (*ϕ*_α1–6_ = 70, *φ*_α1–6_ = 90, *ω*_α1–6_ = 60), extend-a (*ϕ*_α1–6_ = 70, *φ*_α1–6_ = 180, *ω*_α1–6_ = 60) or extend-b (*ϕ*_α1–6_ = 70, *φ*_α1–6_ = 180, *ω*_α1–6_ = 180) according to the definition by Re *et al.*^11^, within the Euclidian distance of 45°.

## Additional Information

**How to cite this article**: Nagae, M. *et al.* Atomic visualization of a flipped-back conformation of bisected glycans bound to specific lectins. *Sci. Rep.* **6**, 22973; doi: 10.1038/srep22973 (2016).

## Supplementary Material

Supplementary Information

## Figures and Tables

**Table 1:** Data collection and refinement statistics of Calsepa and PHA-E bisected glycan complexes.

Data collection statistics		
Crystal ID	Calsepa- glycan 1	PHA-E- glycan 2
Space group	*P*1	*P*2_1_
Cell constants	*a* = 47.6, *b* = 52.8, *c* = 54.8 Å,	*a* = 95.2, *b* = 122.8, *c* = 97.7 Å,
	α = 90.0°, *β* = 90.0°, γ = 94.0°	*β* = 90.7°
Resolution (Å)	100–1.85	100–3.00
	(1.88–1.85)	(3.05–3.00)
*R*_sym_ (%)*	8.8 (45.2)	7.1 (32.0)
Completeness (%)*	97.0 (96.0)	99.9 (100)
Multiplicity*	1.9 (2.0)	4.2 (4.2)
< I >/< σI > *	14.9 (1.9)	23.6 (5.0)
Refinement statistics		
Resolution	54.77–1.85	100–3.00
Unique reflection	41,846	42,772
Number of refined atoms		
Protein	4,335	14,656
Carbohydrate	154	800
Water	207	32^**^
Metal ion	–	16
*R* (%)	24.7	23.1
*R*_free_ (%)	27.9	27.4
Mean *B* value (Å^2^)		
Protein	22.9	41.2
Carbohydrate	27.9	58.9
Water	25.0	43.1
Metal ion	–	45.5
Overall	23.1	42.1
Root mean square deviations from ideal values		
Bond length (Å)	0.006	0.005
Bond angle (°)	1.079	1.053
Ramachandran plot		
Favored (%)	97.0	95.3
Outlier (%)	0	0.05

*Values in the parenthesis are the highest resolution shells.

**All water molecules in PHA-E complex directly coordinate with metal ions.
