# Supplementary material for: Atomic visualization of a flipped-back conformation of bisected glycans bound to specific lectins
Source: Sci Rep. 2016 Mar 14;6:22973. doi: 10.1038/srep22973 (PMC4789653; doi:10.1038/srep22973)
Supplement: Supplementary Information [file srep22973-s1.pdf]

**Supplementary Information**

# Atomic visualization of a flipped-back conformation of bisected glycans bound to specific lectins

Masamichi Nagae[1], Mayumi Kanagawa[1], Kana Morita-Matsumoto[1], Shinya Hanashima[2], Yasuhiko Kizuka[3], Naoyuki Taniguchi[3] and Yoshiki Yamaguchi[1, *]

[1]Structural Glycobiology Team and [3]Disease Glycomics Team, Systems Glycobiology Research Group, RIKEN-Max Planck Joint Research Center, RIKEN Global Research Cluster, 2-1 Hirosawa, Wako, Saitama 351-0198, Japan
[2]Department of Chemistry, Osaka University, Machikaneyama, Toyonaka, Osaka 560-0043, Japan

*Correspondence: yyoshiki@riken.jp

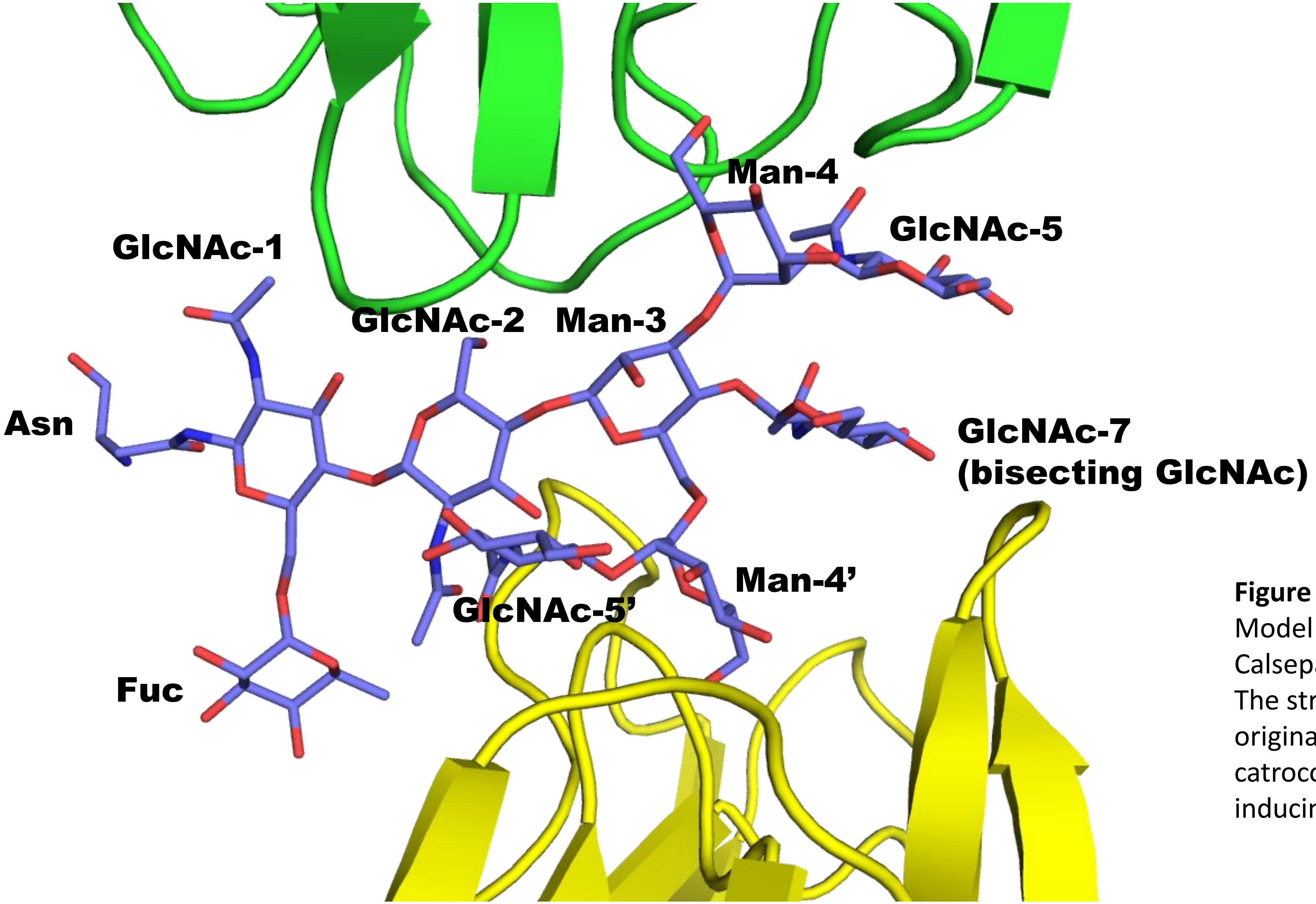


**Figure S1**
Model of the complex between Calsepa and intact bisected *N*-glycan. The structure of chitobiose moiety originates from the crystal structure of catrocollastatin/vascular apoptosis-inducing protein (PDB code: 2DW2).

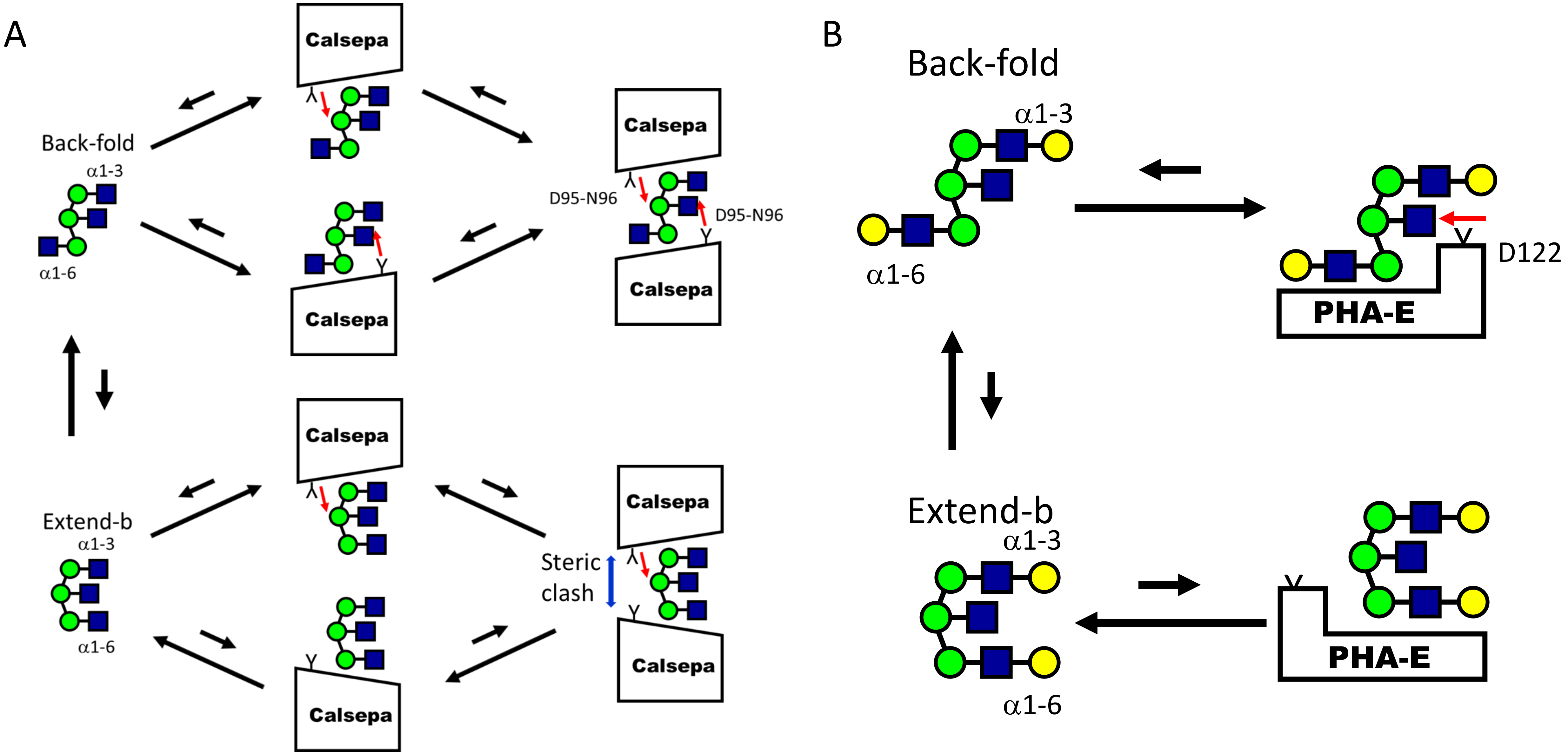


**Figure S2**

Schematic representation of conformer-selective recognition mechanism of Calsepa (A) and PHA-E (B). The specific interactions are indicated with red arrows.

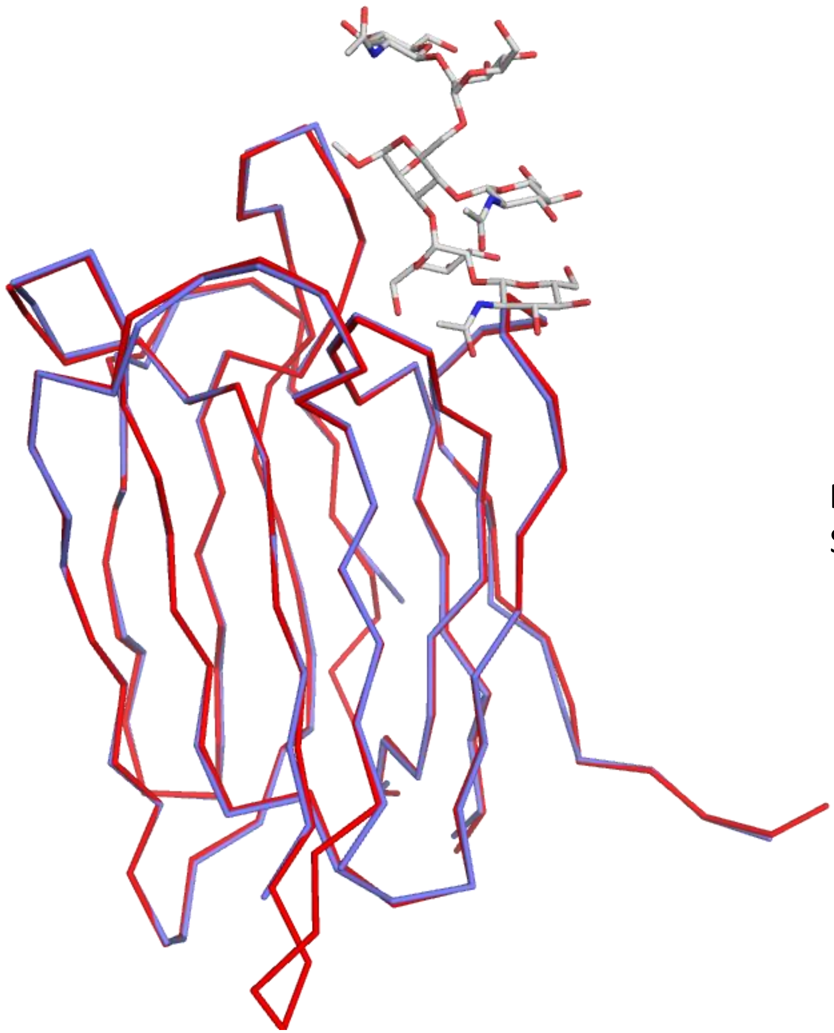

**Figure S3**
Superposition of apo (red) and ligand-bound (blue) Calsepa lectins.

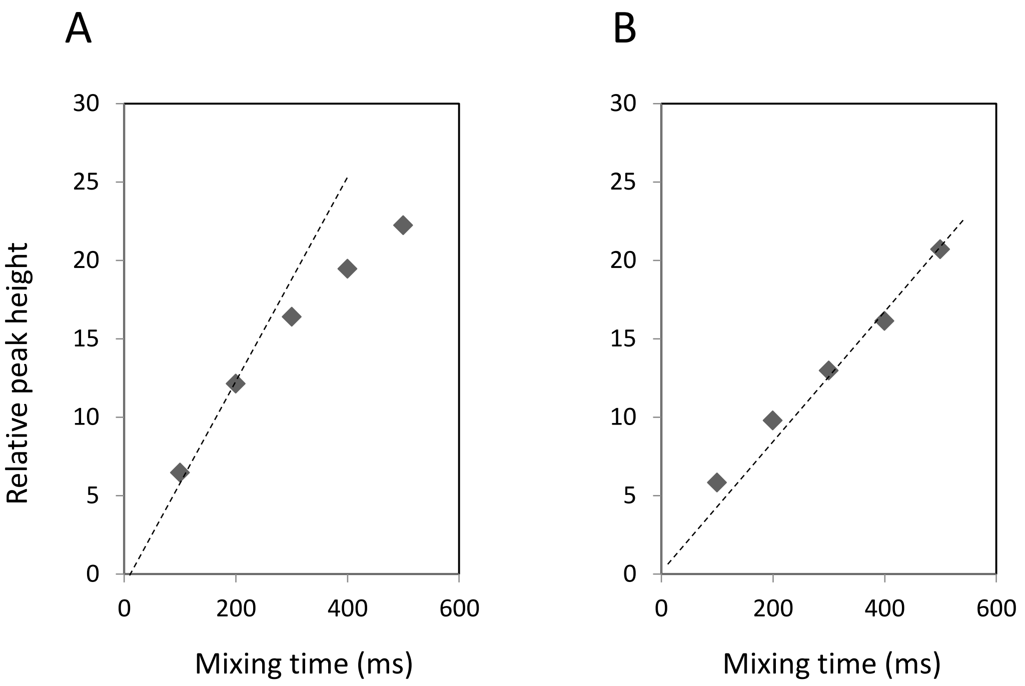


**Figure S4**

NOE build up curves for Man-3 H1 and Man-3 *O*-methyl proton signals in the presence (A) and absence (B) of Calsepa lectin in 1D selective NOESY experiments. Man-3 H1 signal was selectively inverted using 180$^{o}$ rectangular pulse with 40-ms duration.

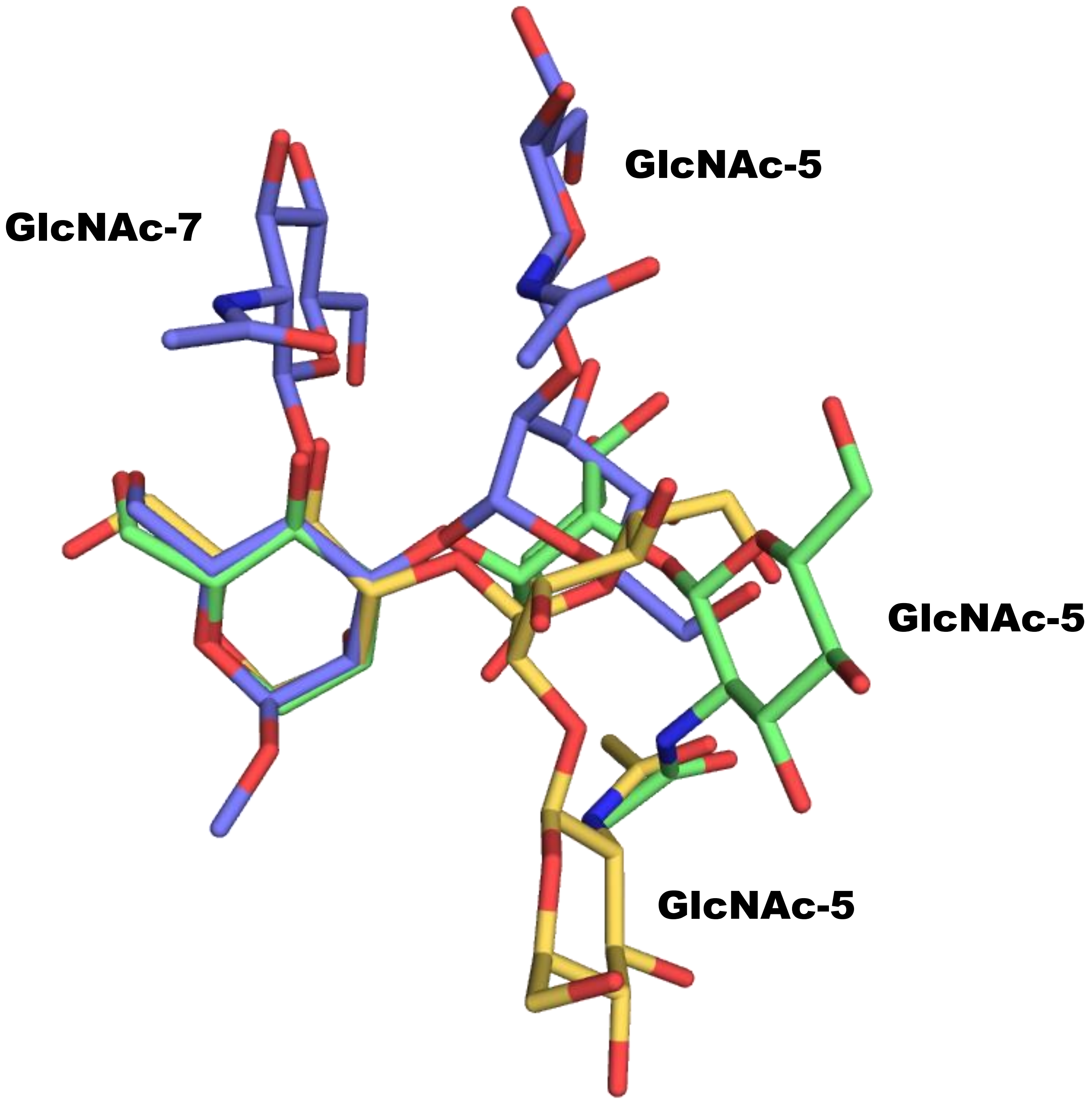


**Figure S5**
Structural superposition of α1-3 branches of bisected glycan (Calsepa lectin; blue) and non-bisected glycans (1SLC_354; light green, 1ZAG_A; brown). The α1-6 branches are omitted for clarity.

**Table S1**

Direct interactions between Calsepa and bisected glycan. Two pairs of Calsepa-bisected glycan complexes exist in the asymmetric unit: molecules A and B of Calsepa and molecule E of bisected glycan, molecules C and D of Calsepa and molecule F of bisected glycan. Hydrogen bond distances of each complex are indicated separately.

| Calsepa | Bisected glycan | Distance (Å) |
|---|---|---|
| Molecule A or C | Molecule E or F | (A, B, E), (C, D, F) |
| Asn96 Oδ1 | Man-3 OH2 | 3.5, 3.1 |
| Gly17 N | Man-4 OH3 | 2.9, 2.9 |
| Asp144 Oδ2 | Man-4 OH4 | 2.6, 2.6 |
| Tyr141 N | Man-4 O5 | 3.1, 3.1 |
| Tyr142 N | Man-4 OH6 | 2.9, 2.9 |
| Asp144 Oδ1 | Man-4 OH6 | 2.7, 2.7 |
| Asn18 Nδ2 | GlcNAc-5 OH3 | 2.7, 2.7 |
| Asp95 Oδ2 | GlcNAc-5' OH4 | 2.8, 3.0 |
| | | |
| Molecule B or D | Molecule E or F | |
| Asp95 Oδ2 | GlcNAc-5 OH4 | 3.3, 3.0 |
| Asp95 O | GlcNAc-7 OH4 | 2.7, 2.8 |
| Asn96 Oδ1 | GlcNAc-7 OH4 | 3.2, 3.3 |
| Gly17 N | Man-4' OH3 | 2.8, 2.9 |
| Asp144 Oδ2 | Man-4' OH4 | 2.7, 2.6 |
| Tyr141 N | Man-4' O5 | 3.0, 3.0 |
| Asp144 Oδ1 | Man-4' OH6 | 2.7, 2.7 |
| Tyr142 N | Man-4' OH6 | 2.8, 2.9 |
| Asn18 Nδ2 | GlcNAc-5' OH3 | 2.6, 2.6 |

**Table S2**

Dihedral angles of bisected glycans in Calsepa complex and PHA-E complex. In the case of the PHA-E complex, the averaged values of eight complexes are shown.

Calsepa-bisect glycan complex

| | Molecule E | Molecule F |
|---|---|---|
| GlcNAcβ1-4Man | | |
| ($\phi_{\beta 1\text{-}4}$, $\varphi_{\beta 1\text{-}4}$) | (-66, 137) | (-60, 139) |
| GlcNAcβ1-2Man (α1-3 branch) | | |
| ($\phi_{\beta 1\text{-}2}$, $\varphi_{\beta 1\text{-}2}$) | (-87, -131) | (-88, -137) |
| GlcNAcβ1-2Man (α1-6 branch) | | |
| ($\phi_{\beta 1\text{-}2}$, $\varphi_{\beta 1\text{-}2}$) | (-92, -130) | (-90, -128) |
| Manα1-3Man | | |
| ($\phi_{\alpha 1\text{-}3}$, $\varphi_{\alpha 1\text{-}3}$) | (103, -146) | (105, -142) |
| Manα1-6Man | | |
| ($\phi_{\alpha 1\text{-}6}$, $\varphi_{\alpha 1\text{-}6}$, $\omega_{\alpha 1\text{-}6}$) | (97, 104, 57) | (98, 107, 57) |

PHA-E-bisect glycan complex (molecule A-H)

| | |
|---|---|
| GlcNAcβ1-4Man | |
| ($\phi_{\beta 1\text{-}4}$, $\varphi_{\beta 1\text{-}4}$) | (-80 ± 4, 124 ± 3) |
| Manβ1-4GlcNAc | |
| ($\phi_{\beta 1\text{-}4}$, $\varphi_{\beta 1\text{-}4}$) | (-64 ± 6, 122 ± 2) |
| GlcNAcβ1-2Man (α1-3 branch) | |
| ($\phi_{\beta 1\text{-}2}$, $\varphi_{\beta 1\text{-}2}$) | (-94 ± 5, -111 ± 5) |
| GlcNAcβ1-2Man (α1-6 branch) | |
| ($\phi_{\beta 1\text{-}2}$, $\varphi_{\beta 1\text{-}2}$) | (-90 ± 2, -104 ± 3) |
| Galβ1-4GlcNAc (α1-6 branch) | |
| ($\phi_{\beta 1\text{-}4}$, $\varphi_{\beta 1\text{-}4}$) | (-59 ± 4, 114 ± 4) |
| Manα1-3Man | |
| ($\phi_{\alpha 1\text{-}3}$, $\varphi_{\alpha 1\text{-}3}$) | (69 ± 4, -121 ± 3) |
| Manα1-6Man | |
| ($\phi_{\alpha 1\text{-}6}$, $\varphi_{\alpha 1\text{-}6}$, $\omega_{\alpha 1\text{-}6}$) | (62 ± 2, 122 ± 5, 54 ± 4) |

**Table S3**

Dihedral angles of bisected and non-bisected biantennary glycans obtained so far. The coordinates of non-bisected and bisected biantennary *N*-glycans are extracted from Protein Data Bank. The ideal conformations of extend-a, extend-b and back-fold are as follows: extend-a (Ex-a) ($\phi_{\alpha 1\text{-}6}$=70, $\varphi_{\alpha 1\text{-}6}$=180, $\omega_{\alpha 1\text{-}6}$=60), extend-b (Ex-b) ($\phi_{\alpha 1\text{-}6}$=70, $\varphi_{\alpha 1\text{-}6}$=180, $\omega_{\alpha 1\text{-}6}$=180), and back-fold (BF) ($\phi_{\alpha 1\text{-}6}$=70, $\varphi_{\alpha 1\text{-}6}$=90, $\omega_{\alpha 1\text{-}6}$=60). The conformation of each glycan is judged from the "distance" from ideal values of less than 45°. For clarity, the three angles of the α1-6 linkage are described as 0~360° instead of -180~+180°.

(i) Non-bisected biantennary glycan

| | | Glycan on glycoproteins | | | | | | Lectin or antibody complex | | | | | | | | | | | |
|---|---|---|---|---|---|---|---|---|---|---|---|---|---|---|---|---|---|---|---|
| PDB code | | 1ZAG_A | 1ZAG_B | 1L6X | 4JS1 | 3VUN | 1CVI | 1LGB | 1LGC | 1TEI | 2ARX | 3ZYR | 1K9I | 1SLB_354 | 1SLB_364 | 1SLC_354 | 1SLC_364 | 4FQC | 2VUZ |
| Manα1−3Man | $\phi_{\alpha 1\text{-}3}$ | 173 | 76 | 73 | 74 | 94 | 71 | 81 | 93 | 62 | 72 | 71 | 65 | 88 | 96 | 88 | 74 | 68 | 79 |
| | $\varphi_{\alpha 1\text{-}3}$ | −60 | −106 | −101 | −118 | −114 | −146 | −101 | −113 | −99 | −106 | −110 | −106 | −141 | −105 | −54 | −108 | −88 | −114 |
| Manα1−6Man | $\phi_{\alpha 1\text{-}6}$ | 116 | 192 | 68 | 94 | 276 | 194 | 61 | 36 | 170 | 85 | 90 | 52 | 194 | 91 | 72 | 147 | 45 | 54 |
| | $\varphi_{\alpha 1\text{-}6}$ | 205 | 253 | 174 | 169 | 216 | 236 | 198 | 151 | 196 | 212 | 207 | 166 | 260 | 202 | 181 | 164 | 198 | 90 |
| | $\omega_{\alpha 1\text{-}6}$ | 179 | 206 | 179 | 174 | 164 | 165 | 72 | 184 | 157 | 68 | 62 | 64 | 176 | 212 | 49 | 51 | 200 | 22 |
| GlcNAcβ1−2Man (α1−3 branch) | $\phi_{\beta 1\text{-}2}$ | −108 | −137 | −95 | −90 | −88 | −61 | 53 | 49 | −80 | −84 | −85 | −79 | −91 | −75 | −100 | −91 | −82 | −55 |
| | $\varphi_{\beta 1\text{-}2}$ | −97 | 32 | −82 | −112 | −84 | −6 | −89 | −115 | −82 | −74 | −88 | −93 | −83 | −59 | −89 | −106 | −91 | −114 |
| GlcNAcβ1−2Man (α1−6 branch) | $\phi_{\beta 1\text{-}2}$ | −88 | −115 | −80 | −105 | −81 | 29 | 41 | 63 | −71 | 76 | −79 | −68 | −85 | −86 | −98 | −90 | −94 | −70 |
| | $\varphi_{\beta 1\text{-}2}$ | −87 | −94 | −85 | −122 | −91 | −150 | −123 | −87 | −132 | −92 | −124 | −78 | −128 | −79 | −86 | −105 | −93 | −111 |
| Conformation | | | | Ex−a | Ex−b | | | Ex−a | Ex−b | | Ex−a | Ex−a | Ex−a | | Ex−b | Ex−a | | Ex−a | BF |

(ii) Bisected biantennary glycan

| | | Glycan on glycoprotein | | Lectin complex | | |
|---|---|---|---|---|---|---|
| PDB code | | 2DW2 | 3SGK | 3VYK | Calsepa | PHA−E |
| Manα1−3Man | $\phi_{\alpha 1\text{-}3}$ | 71 | 87 | 76 | 104 | 69 |
| | $\varphi_{\alpha 1\text{-}3}$ | −145 | −119 | −120 | −144 | −121 |
| Manα1−6Man | $\phi_{\alpha 1\text{-}6}$ | 67 | 80 | 66 | 98 | 62 |
| | $\varphi_{\alpha 1\text{-}6}$ | 128 | 170 | 193 | 106 | 122 |
| | $\omega_{\alpha 1\text{-}6}$ | 51 | 192 | 204 | 57 | 54 |
| GlcNAcβ1−2Man (α1−3 branch) | $\phi_{\beta 1\text{-}2}$ | −59 | −91 | −78 | −88 | −94 |
| | $\varphi_{\beta 1\text{-}2}$ | 74 | −81 | −88 | −134 | −111 |
| GlcNAcβ1−2Man (α1−6 branch) | $\phi_{\beta 1\text{-}2}$ | −82 | −94 | −77 | −91 | −90 |
| | $\varphi_{\beta 1\text{-}2}$ | −98 | −86 | −81 | −129 | −104 |
| GlcNAcβ1−4Man | $\phi_{\beta 1\text{-}4}$ | −81 | −47 | −85 | −63 | −80 |
| | $\varphi_{\beta 1\text{-}4}$ | 135 | 132 | 130 | 138 | 124 |
| Conformation | | BF | Ex−b | Ex−b | BF | BF |
